# Essential Oil from *Piper aduncum*: Chemical Analysis, Antimicrobial Assessment, and Literature Review

**DOI:** 10.3390/medicines4030049

**Published:** 2017-07-02

**Authors:** Lianet Monzote, Ramón Scull, Paul Cos, William N. Setzer

**Affiliations:** 1 Parasitology Department, Institute of Tropical Medicine Pedro Kouri, 10400 Havana, Cuba; monzote@ipk.sld.cu; 2 Department of Chemistry, Institute of Pharmacy and Food, University of Havana, 13600 Havana, Cuba; rscull@ifal.ih.cu; 3 Laboratory for Microbiology, Parasitology and Hygiene, Faculty of Pharmaceutical, Biomedical and Veterinary Sciences, Antwerp University, 2610 Wilrijk, Belgium; paul.cos@uantwerpen.be; 4 Department of Chemistry, University of Alabama in Huntsville, Huntsville, AL 35899, USA

**Keywords:** *Piper aduncum*, essential oil, cluster analysis, chemotypes, antimicrobial, parasitic protozoa

## Abstract

**Background:** The challenge in antimicrobial chemotherapy is to find safe and selective agents with potency that will not be compromised by previously developed resistance. Terrestrial plants could provide new leads to antibacterial, antifungal, or antiprotozoal activity. **Methods:** The essential oil (EO) of *Piper aduncum* L. (Piperaceae) from Cuba was analyzed by gas chromatography—mass spectrometry (GC-MS). A cluster analysis of *P. aduncum* EO compositions reported in the literature was carried out. The EO was screened against a panel of microorganisms (bacteria, fungi, parasitic protozoa) as well as for cytotoxicity against human cells. In addition, a review of scientific literature and a bibliometric study was also conducted. **Results:** A total of 90 compounds were identified in the EO, of which camphor (17.1%), viridiflorol (14.5%), and piperitone (23.7%) were the main components. The cluster analysis revealed at least nine different chemotypes. The EO did not show notable activity against bacteria or fungi, but was active against parasitic protozoa. **Conclusions:** The results from this study indicate *P. aduncum* from Cuba is a unique chemotype, support the importance of *P. aduncum* EOs as medicines, and demonstrate the promise of Cuban *P. aduncum* EO as a chemotherapeutic agent against parasitic protozoal infections.

## 1. Introduction

The challenge in antimicrobial chemotherapy is to find safe and selective agents with potency that will not be compromised by previously developed resistance. In this context, the search for new antimicrobial compounds from terrestrial plants could provide new leads to antibacterial, antifungal, or antiprotozoal activity. The World Health Organization also advocates the use of natural medicines for the treatment of microbial infectious agents. The search for new medicinal agents has become extremely important, and higher plants are a very rich source of new and selective substances with therapeutic potential against these ailments. There are approximately 250,000 plant species worldwide, of which only a fraction has so far been studied [1,2].

**Figure 1:**
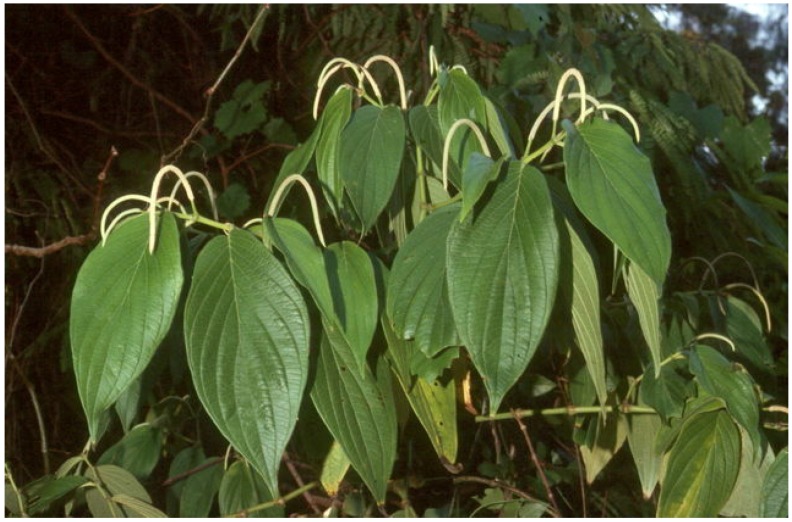
Photograph of *Piper aduncum* L. (© Copyright Bobby Hattaway, 2011 [10]).

The Piperaceae family is distributed in tropical and subtropical regions in the northern and southern hemispheres and comprises around 3600 species. The *Piper* genus constitutes the most represented, with around 2000 species [3]. In particular, biological properties of essential oils (EOs) from *Piper aduncum* L. (Figure 1) have been widely studied, including antiprotozoal [4,5], anthelminthic [6], antioxidant [7], and insecticidal [8,9] activities. Considering these previously documented effects and the potentialities as therapeutic agents, we have focused on the EO from *P. aduncum* growing in Cuba. In this sense, chemical characterization of the EO was performed and the EO was also screened against a selection of microorganisms, including a Gram-negative (*Escherichia coli*) and a Gram-positive (*Staphylococcus aureus*) bacterium, a yeast (*Candida albicans*), and protozoa that are responsible for human parasitic infections: malaria (*Plasmodium falciparum*), sleeping sickness (*Trypanosoma brucei*), Chagas disease (*T. cruzi*), cutaneous (*Leishmania amazonensis*) and visceral (*L. donovani* and *L. infantum*) leishmaniasis. For comparison, in vitro cytotoxicity screening was performed on MRC-5 (normal human lung) cells. Finally, a summary of relevant aspect in the cited scientific literature of EO from *P. aduncum* was reviewed and a bibliometric study was also conducted.

## 2. Materials and Methods

### 2.1. Plant Material

Aerial parts of *P. aduncum* were collected in the early morning of June 2010 from the Institute of Pharmacy and Food, Havana, Cuba. The plant was identified by M.Sc. Ramón Scull following the guidelines of the National Flora of Cuba; a voucher specimen was deposited in the Experimental Station of Medicinal Plants “Dr. Juan Tomás Roig”, Mayabeque, Cuba, under registration voucher number 4649. The fresh plant sample was manually crushed and the EO was immediately hydrodistilled by the conventional method using a Clevenger type apparatus for 5 h, the oil was separated, and stored in an amber-colored glass bottle under refrigeration (4 °C) prior to analysis. Solutions of the EO in dimethylsulfoxide (DMSO) were prepared (20 mg/mL) and used for biological evaluations (see below).

### 2.2. Gas Chromatography—Mass Spectrometry

Chemical characterization of the EO from *P. aduncum* was performed by gas chromatography/mass spectrometry using an Agilent 6890 gas chromatography (Agilent Technologies, Palo Alto, CA, USA) coupled with an Agilent 5973 mass selective detector (MSD) (Agilent Technologies, Palo Alto, CA, USA), operated in the electron impact (EI) mode with electron energy = 70 eV, a scan range of 40–400 amu, and a scan rate of 3.99 scans/s, and operated through an Agilent ChemStation data system (G1701CA, version C.00.01.080, Agilent Technologies, Palo Alto, CA, USA). The GC column was an HP-5ms fused silica capillary column (Agilent Technologies, Palo Alto, CA, USA), with (5% phenyl)-polymethylsiloxane stationary phase, a film thickness of 0.25 μm, a length of 30 m, and an internal diameter of 0.25 mm. The carrier gas was helium with a column head pressure of 48.7 kPa and a flow rate of 1.0 mL/min. The inlet temperature was 200 °C, the interface temperature was 280 °C, and the following GC oven temperature program was used: 40 °C initial temperature, which was held for 10 min; increased at 3 °C/min to 200 °C; increased 2 °C/min to 220 °C. A 1% *w*/*v* solution of the EO in CH_2_Cl_2_ was prepared and 1 μL was injected using a splitless injection technique. Identification of the EO components was achieved using their retention indices (calculated relative to a homologous series of normal alkanes, and by comparison of their mass spectral fragmentation patterns with those reported in the literature [11] and our own in-house database. The percentages of the constituents were calculated from total ion current without standardization.

### 2.3. Hierarchical Cluster Analysis

A total of 38 EO compositions of *P. aduncum* from the published literature, including the EO chemical makeup from this work, were treated as operational taxonomic units (OTUs). The percentages of 35 main essential oil compounds (α-pinene, β-pinene, myrcene, α-terpinene, limonene, 1,8-cineole, (*Z*)-β-ocimene, (*E*)-β-ocimene, terpinolene, linalool, camphor, borneol, terpinen-4-ol, α-terpineol, piperitone, α-copaene, β-caryophyllene, aromadendrene, α-humulene, germacrene D, valencene, asaricin, bicyclogermacrene, γ-cadinene, myristicin, δ-cadinene, *epi*-longipinanol, (*E*)-nerolidol, longipinanol, spathulenol, caryophyllene oxide, viridiflorol, dill apiole, τ-cadinol, and α-cadinol) were used to determine the chemotypic characteristics of the various *P. aduncum* essential oil compositions by agglomerative hierarchical cluster (AHC) analysis using the XLSTAT software, version 2015.4.01 (Addinsoft™, New York, NY, USA). To determine similarity, the Pearson correlation was used, and the clusters were defined using the unweighted pair-group method with arithmetic average (UPGMA).

### 2.4. Bioactivity Evaluations

#### 2.4.1. Reference Drugs

Chloramphenicol and erythromycin were obtained from Sigma-Aldrich (Bornem, Belgium), while miconazole was generously donated by Janssen Pharmaceuticals (Beerse, Belgium). Benznidazol, chloroquine, miltefosine, and suramine were kindly donated by WHO-TDR and amphotericin B from Imefa (La Habana, Cuba).

#### 2.4.2. Microorganisms and Cell Culture

Microorganisms used were: *E. coli* ATCC8739, *S. aureus* ATCC6538, *C. albicans* B59630, *P. falciparum* Ghana, *T. b. brucei* Squib-427, *T. cruzi* Tulahuen CL2, *L. amazonensis* MHOM/77BR/LTB0016, *L. donovani* MHOM/ET/67/L82, and *L. infantum* MHOM/MA(BE)/67. Cytotoxicity was tested against the mammalian cell line MRC-5 SV2 from the European Collection of Cell Cultures.

#### 2.4.3. Antimicrobial Tests

The integrated panel of microbial screens for the present study and the standard screening methodologies were adopted as described by Cos et al. [12].

*Escherichia coli* and *S. aureus* were cultured in Mueller Hinton broth (MHB) medium, while *C. albicans* was cultured in RPMI medium. Assays were carried out by adding 5 × 10^3^ CFU/well. After 17 h incubation with different EO concentrations at 37 °C, bacterial or fungal viability was determined fluorimetrically after adding resazurin [13] for 30 min at 37 °C to *E. coli* and *S. aureus* or 4 h at 37 °C to *C. albicans*. Fluorescence was measured using a GENios Tecan fluorimeter (Tecan Group Ltd., Männedorf, Switzerland) (excitation 530 nm, emission 590 nm).

*Plasmodium* parasites were cultured in human erythrocytes A+ at 37 °C under an atmosphere of 3% O_2_, 4% CO_2_, and 93% N_2_ [14] in RPMI-1640 culture medium, supplemented with 0.5 (g/v)% AlbumaxTM. Suspensions of infected human red blood cells (1% parasitemia, 2% hematocrit) were added to each well with the test EO and incubated for an additional 72 h. The plate was then frozen at −20 °C and parasite multiplication was measured by the Malstat method [15]. One hundred microliters of Malstat^TM^ reagent was transferred in a new plate and mixed with 20 µL of the hemolysed parasite suspension for 15 min at room temperature. Then, 20 µL of nitro blue tetrazolium chloride (NBT) at 2 mg/mL/PES at 0.1 mg/mL solution was added and the plate was incubated again for 2 h at room temperature in the dark. Absorbance was read at 655 nm in a Bio-Rad 3550-UV microplate reader (Bio-Rad Laboratories, Hercules, CA, USA).

Antitrypanosomal activity was performed using trypomastigotes of *T. brucei,* cultured at 37 °C and 5% CO_2_ in Hirumi-9 medium [16], supplemented with 10% inactivated fetal calf serum (FCSi). Assays were performed by adding 1.5 × 10^4^ trypomastigotes/well. After 72 h incubation, parasite growth was assessed fluorimetrically by adding resazurin for 24 h at 37 °C. In parallel, the EO at different concentrations was added to 4 × 10^4^ amastigotes of *T. cruzi* in 4 × 10^3^ MRC-5 cells maintained in minimal essential medium (MEM) supplemented with 20 mM l-glutamine, 16.5 mM sodium bicarbonate, and 5% FCSi. After incubation at 37 °C and 5% CO_2_ for 7 days, parasite growth was assessed by adding the β-galactosidase substrate chlorophenol red β-d-galactopyranoside [17] for 4 h at 37 °C and the color reaction was read at 540 nm.

Antileishmanial activity against the promastigote form of *L. amazonensis* and *L. donovani* were performed with exponentially growing cells (10^5^ promastigotes/mL in 199 μL) in 96-well plates. One microliter of EO at different concentrations were added and then incubated at 26 °C for 72 h. After 72 h of incubation, 20 µL of *p*-nitrophenyl phosphate at 20 mg/mL and dissolved in 1 M sodium acetate buffer, 1% Triton X-100, pH 5.5 were added to each well and the plate was incubated at 37 °C for 3 h. The absorbance was determined in an EMS Reader MF Version 2.4-0 (Labsystems Oy, Helsinki, Finland) at a wavelength of 405 nm [18]. In the case of experiment with *L. infantum,* amastigotes were obtained from an infected hamster and were used to infect primary peritoneal mouse macrophages (PMM). Then, 3 × 10^4^ macrophages were transferred to each well of a 96-well plate, and the plate was incubated for 48 h at 37 °C and 5% CO_2_. The cells were then washed and infected with *L. infantum* amastigotes at a density of 15 parasites per cell. After 2 h of infection, pre-diluted concentrations of EO were added to the plates, which were then incubated for an additional 120 h under the same conditions. The supernatant was then discarded, the cells were fixed with methanol, and stained with 10% Giemsa for microscopic analysis. The total parasite burden (equal to the average number of amastigotes per cell) in treated wells was determined for each well.

#### 2.4.4. Cytotoxicity Assay

MRC-5 cells were cultivated in MEM, supplemented with l-glutamine (20 mM), 16.5 mM sodium hydrogen carbonate, and 5% FCSi at 37 °C and 5% CO_2_. For the assay, 10^4^ MRC-5 cells/well were seeded onto the test plates containing the pre-diluted EO at different concentrations and incubated at the same conditions for 72 h. Cell viability was determined fluorimetrically 72 h after the addition of resazurin, as previously described (see above).

#### 2.4.5. Statistical Analysis

In each case, the percentage growth inhibition for each concentration of EO was calculated compared to the untreated controls. The median inhibitory (*IC*_50_) and median cytotoxicity (*CC*_50_) concentration values were determined from the lineal concentration-response curves. Statistical differences between values were determined using Mann-Whitney with Statistica for Windows Program, Release 4.5, 1993 (StatSoft, Inc., Tulsa, OK, USA).

### 2.5. Literature Review

#### 2.5.1. Bibliometric Study

With the objective of obtaining reports about EOs from *P. aduncum*, an electronic search in PubMed [19] database was performed. Online access was on 30 January 2017 and the study period was from 1 January 1945 to 30 January 2017. The subject content analysis of records was conducted according to the Medical Subject Headings (MeSH), using the MeSH terms or descriptors ‘Piper aduncum’ and ‘essential oil’. We did not restring any language or document type, in order to analyze publication patterns of all publications in the studied thematic. 

The number of papers by year was obtained, as well as the language used and journals according to main topic. In addition, productivity by country was analyzed, considering only the institutional affiliation for the first participating author.

#### 2.5.2. Pharmacological and Chemical Reports

The scientific reports previously obtained were screened and analyzed to identify and summarize the pharmacological and chemical studies of EOs from *P. aduncum* as well. The data extracted from the literature included country of collection, pharmacological application, and main chemical isolated compounds. In this case, there were also no exclusions related to publication dates or languages.

## 3. Results and Discussion

### 3.1. Chemical Analysis of the Essential Oil from Piper aduncum

**Figure 2:**
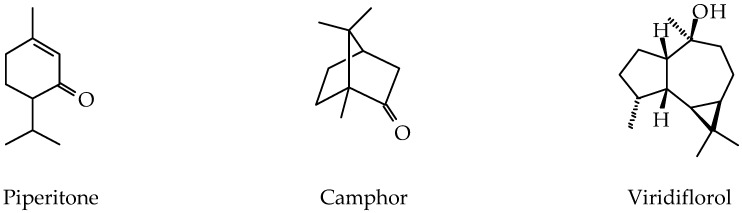
Chemical structures of the major components identified in the essential oil from *Piper aduncum* L. collected in Havana, Cuba.

A total of 90 compounds (Table 1) were found in the chromatogram of the EO obtained by gas chromatography coupled with mass spectrometry (GC-MS). The essential oil of *P. aduncum* from Cuba was dominated by oxygenated monoterpenoids (50.3%) and oxygenated sesquiterpenoids (29.2%), followed by monoterpene hydrocarbons (9.7%) and sesquiterpene hydrocarbons (8.2%), and an unidentified diterpenoid (2.0%). Piperitone, camphor, and viridiflorol (Figure 2) were the main components with 23.7%, 17.1%, and 14.5%, respectively.

**Figure 3:**
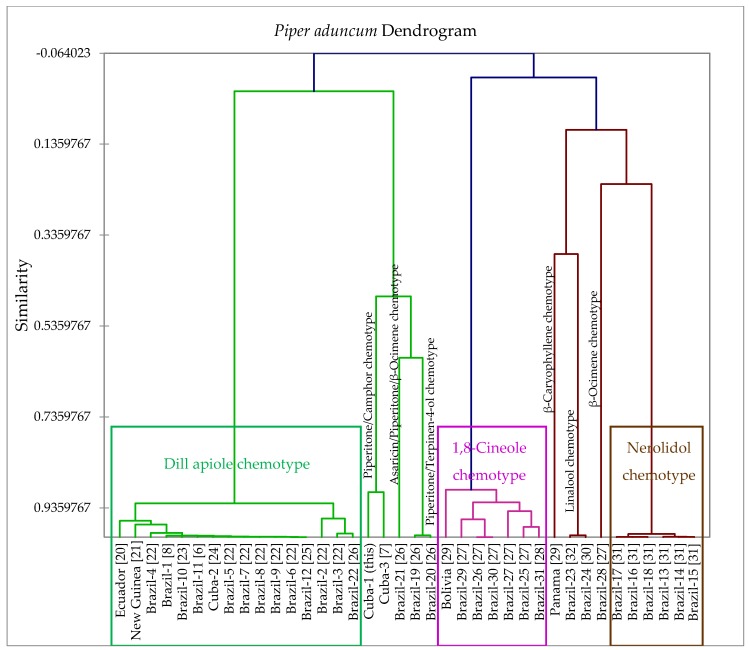
Dendrogram acquired from the agglomerative hierarchical cluster analysis of 38 *Piper aduncum* essential oil compositions.

In perusing the *P. aduncum* essential oil literature [7,8,20,21,22,23,24,25,26,27,28,29,30,31,32], there is a large degree of variation in compositions of the essential oils. Therefore, a hierarchical cluster analysis of the essential oil compositions was carried out (Figure 3). From the cluster analysis, we can define several different chemotypes: (1) a dill apiole chemotype; (2) a 1,8-cineole chemotype; (3) a (*E*)-nerolidol chemotype; (4) a linalool chemotype; (5) a β-caryophyllene chemotype; (6) a (*E*)-β-ocimene chemotype, and three mixed chemotypes rich in piperitone; (7) a piperitone/camphor chemotype; (8) a piperitone/terpinen-4-ol chemotype; and (9) an asaricin/piperitone/(*E*)-β-ocimene chemotype. The sample from Cuba in this work belongs to the piperitone/camphor chemotype and is similar in composition to another *P. aduncum* sample from Cuba [7]. There does seem to be some geographical correlation with chemotype. For example, the samples from the same region of northeastern Brazil all belong to the nerolidol chemotype [31]. Although we cannot rule out bias based on compound identification, quantitation, or detection, it is unlikely to significantly affect the cluster; dill apiole, 1,8-cineole, and nerolidol are readily identifiable essential oil components.

### 3.2. Antimicrobial Screening

Several studies have indicated that EOs may be an antimicrobial alternative to antibiotics. In this sense, a wide panel of microorganisms that causes infectious diseases was included. In our study, the reference drugs caused a potent inhibition, as was expected, while different activities of *P. aduncum* EO were observed against the microorganisms tested. In the antibacterial and antifungal assays, the EO did not exhibit activity at 64 µg/mL (the highest concentration used), except against *S. aureus*, which did show some activity (Table 2). Currently, one of the widely used applications of EOs includes food preservatives, precisely due to their bactericidal and fungicidal properties [33,34,35]. However, the EO from *P. aduncum* in this work did not display high activity against human pathogenic bacteria and fungi.

In contrast, against protozoal parasites, the *P. aduncum* EO was able to inhibit all pathogens (Table 3). Parasitic protozoal infections cause much human morbidity and mortality. The prevalence of these diseases is higher in the tropics, where a significant number of deaths is attributed to malaria, leishmaniasis, African and American trypanosomiasis [36]—protozoal parasites that are included in this study. The best antiprotozoal activity of *P. aduncum* EO was observed against *P. falciparum*, the causal agent of malaria. Discovering new drugs in this field is a health priority because malaria is the major parasitic infection in many tropical and subtropical regions. Currently, more than one million deaths occur each year, and between 400 and 500 million new cases are reported annually; it is estimated that 40% of the world’s population is at risk of malaria [37].

In addition to the *Plasmodium* parasite, flagellated parasitic protozoa belonging to the genera *Trypanosoma* and *Leishmania* (Trypanosomatidae) were also included, which are considered Neglected Tropical Diseases (NTDs) by the World Health Organization [38]. In this sense, *T. brucei* is a causative agent of African trypanosomiasis or sleeping sickness, while *T. cruzi* causes American trypanosomiasis, commonly known as Chagas disease. Leishmaniasis exhibits a range of clinical appearances including cutaneous (CL), mucocutaneous (MCL), and visceral (VL) forms. In these human pathogenic trypanosomes, the management of the infections is currently based on chemotherapeutics that are not ideal due to cost, availability, toxicity, and resistance [39]. 

Among trypanosomes, both species of *Trypanosoma* were inhibited at the same concentration range with IC_50_ values around 2 µg/mL. African trypanosomiasis invariably leads to coma and death if left untreated, with two stages. The first phase is characterized by nonspecific clinical symptoms, and trypanosomes are restricted to the lymphatic and circulatory systems, whereas in the second stage severe neurological symptoms appear, and parasites can be found in the brain and in cerebrospinal fluid [40]. It has been estimated that 60 million people in sub-Saharan Africa countries are currently at risk, and around 50,000 to 70,000 people are infected [39]. Chagas disease is present in Central and South America, with an annual death toll of about 50,000 people in 18 endemic countries, while more than 8 million people are infected, while nearly 90 million people are at risk of infection [41]. Recently, Villamizar and collaborators also demonstrated the antitrypanosomal activity of EO from *P. aduncum* collected in Brazil, showing an *IC*_50_ of 2.8, 12.1, and 9 μg/mL against cell-derived, metacyclic trypomastigotes, and intracellular amastigotes, respectively [5]. In addition, they suggested that the probable mechanism of action was related with decreases in the mitochondrial membrane potential of the parasite after treatment [5].

*Leishmania* spp. showed variable susceptibilities to *P. aduncum* EO, with the species that cause visceral leishmaniasis (*L. infantum* and *L. donovani*) showing lower *IC*_50_ values. In the clinical form of VL, the parasites colonize the bone marrow, liver, and spleen, resulting in host immunosuppression and death in the absence of treatment [39]. Finally, the species that causes CL (*L. amazonensis*), in which parasites remain localized in the epithelial tissues, the EO displayed a higher *IC*_50_ value. Currently, leishmaniasis affects 12 million people in 98 countries where the disease is endemic and 350 million people live at risk of infection. In particular, the number of cases of VL is calculated to be as high as 0.2–0.4 million people per year, with mortality estimated at 10–20%, especially in poor areas [42]. Notably, Bernuci and collaborators reported that the EO from *P. aduncum* growing in Brazil displayed activity against *L. amazonensis* [43].

The antiprotozoal potential of *P. aduncum* essential oil has been demonstrated in this study. The activity may be attributed to the major components piperitone, camphor, and viridiflorol. Both piperitone and camphor have shown antiprotozoal activity against the bloodstream forms of *Trypanosoma brucei brucei* [44,45]. To our knowledge, however, there have been no reports on the antiprotozoal activity of viridiflorol. Nevertheless, the antiprotozoal activity of the Cuban *P. aduncum* chemotype EO shows promise, complements the reports of activity for other chemotypes of *P. aduncum*, and underscores the importance of EOs as potential treatment options for neglected tropical diseases.

In this present study, the cytotoxicity of *P. aduncum* EO was also studied using MRC-5 (Human fetal lung fibroblast) cells as a model. The EO inhibited cell growth, with an *IC*_50_ value of 5.1 µg/mL. Other authors also reported the cytotoxicity of EO from *P. aduncum*, in particular the EO collected in Brazil [5,43]. In this study, the reference drug tamoxifen also showed a high cytotoxic effect (*IC*_50_ = 4.3 µg/mL).

### 3.3. Literature Review

#### 3.3.1. Bibliometric Study

To carry out the bibliometric study we used the PubMed database, which is one of the most used databases by academics, medical students, and primary care practitioners according to several surveys. In addition, it is easily accessible and widely used, and it uses a controlled vocabulary for indexing and recovering documents with a certain criterion for quality [46,47,48,49].

**Figure 4:**
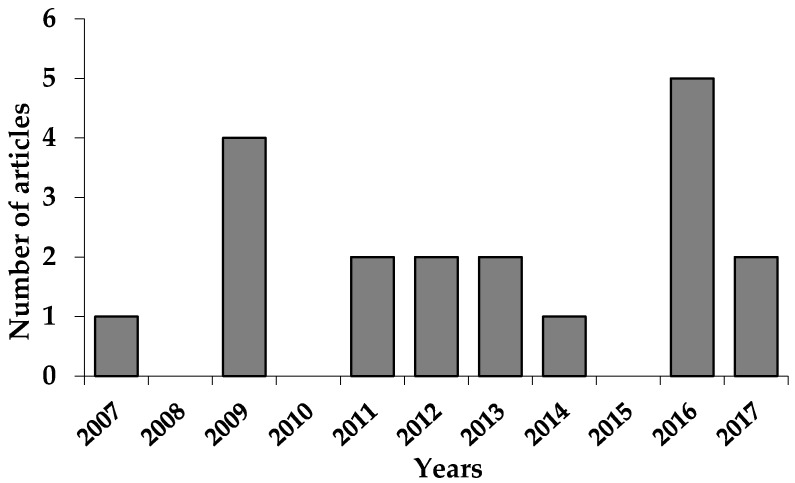
Number of articles found in *PubMed* database regarding essential oils from *Piper aduncum* L. by year (*n* = 19).

A total of 20 articles were obtained, of which one was repeated, thus resulting in a total of 19 original reports about studies of EO from *P. aduncum*. Results in this field appear in the last decade, with a particular increase after 2010 (Figure 4). The language used was English in 15 scientific journals. The most used journal was *Pest Management Science* with three articles (16%) followed by *Molecules* and *Chemistry & Biodiversity* with two articles (11%). The remaining journals published only one article each (5%): *Annals of Academia Brasileira Ciencia, Bulletin of Entomological Research, Experimental and Applied Acarology, Iranian Journal of Arthropod-Borne Diseases, Journal of the American Mosquito Control Association, Memórias do Instituto Oswaldo Cruz, Natural Product Communications, Neotropical Entomology, Pharmaceutical Biology, Revista Brasileira de Parasitologia Veterinária, Tropical Biomedicine and Veterinary Parasitology.* The source journals mainly include the fields of vectors > biology > parasitology > biomedicine. The first author’s institutional address displayed that only four countries are involved in these studies, with different research groups. Brazil was the predominant country (63%), followed by Cuba and Malaysia (16%), and finally New Guinea (5%).

This study has shown an increase in the number of publications on EOs from *P. aduncum* in recent years. A high representation in journals related to control of vectors was observed, which is in concordance with the main applications reported for *Piper* essential oils. In addition, the most representative country was Brazil, probably due to the exuberant flora, traditional use of natural products and the technical development of several research groups in the country in the field of chemistry and pharmacology. The bibliometric methodology used may present some limitations and further databases such as Science Direct and Scielo could be analyzed to search for additional studies. Nevertheless, this study represents a useful tool for scientists that are planning to study the EO from *P. aduncum* and could be used to help organize research in the field of EO and natural products in general.

#### 3.3.2. Pharmacological and Chemical Reports

The earlier analyses of scientific reports about the EO from *P. aduncum* indicated that insecticidal and antiparasitic activities have been the most reported of plants collected in tropical countries (Table 4). In the case of vectors, useful applications were found against *Aedes aegypti* mosquitoes, the vector of dengue and yellow fever. Antiparasitic effects were more studied against kinetoplastids, including *Trypanosoma* and *Leishmania*.

Several different components have been identified in EOs from *P. aduncum.* In general, between 23 and 68 compounds have been reported, with a higher percentage of monoterpenes. Unfortunately, many pharmacological studies did not include a chemical characterization of the EO. Some chemical compounds have been identified, isolated, and studied (Table 5). The most representative was the dill apiole, a phenylpropanoid, which showed antifungal, insecticidal, and anti-inflammatory activities.

## 4. Conclusions

The results of this study support the importance of the EO from *P. aduncum*, in particular in regard to antiparasitic activity. Its effect against *P. falciparum* indicates that this EO could be a promising antimalarial agent. Further studies including in vivo bioassays are needed to validate the in vitro results and to ascertain the safety of the EO. In addition, the antimicrobial evaluation of the major components, camphor, viridiflorol, and piperitone, will be useful and will surely provide complementary data about antiparasitic active compounds as well as the mechanism(s) of action. There is significant chemical diversity in the EOs of *P. aduncum*, and this, no doubt, will affect the biological activities. Thus, there is a need to characterize the EOs of this plant and correlate this information with bioactivities.

## Figures and Tables

**Table 1:** Chemical composition of essential oil from *Piper aduncum* L. collected in Havana, Cuba.

RI	Compound	%		RI	Compound	%
789	3-Methyl-2-butenal	tr		1337	δ-Elemene	0.1
858	(3*Z*)-Hexenol	tr		1349	α-Cubebene	tr
932	Tricyclene	tr		1366	Cyclosativene	0.1
942	α-Pinene	0.8		1371	α-Ylangene	0.1
954	Camphene	5.9		1376	α-Copaene	0.5
979	β-Pinene	0.4		1384	β-Bourbonene	tr
989	6-Methyl-5-hepten-2-one	tr		1393	β-Elemene	1.2
993	Myrcene	0.1		1419	β-Funebrene	tr
1005	α-Phellandrene	tr		1420	α-Santalene	0.2
1025	*p*-Cymene	1.0		1424	Carvone hydrate	tr
1028	Limonene	1.5		1429	β-Copaene	0.2
1031	1,8-Cineole	0.1		1436	α-*trans*-Bergamotene	tr
1072	*cis*-Linalool oxide (furanoid)	0.1		1439	Aromadendrene	0.8
1083	Camphenilone	tr		1453	α-Humulene	tr
1088	Fenchone	0.1		1461	β-Santalene	0.5
1089	*trans*-Linalool oxide (furanoid)	0.1		1464	α-Acoradiene	tr
1101	Linalool	1.2		1475	α-Neocallitropsene	tr
1121	*cis-p*-Menth-2-en-1-ol	tr		1478	γ-Muurolene	1.0
1126	α-Campholenal	tr		1481	γ-Curcumene	0.1
1136	*cis-p*-Mentha-2,8-dien-1-ol	tr		1484	*ar*-Curcumene	0.4
1139	*trans*-Pinocarveol	0.1		1487	β-Selinene	0.6
1140	*trans-p*-Menth-2-en-1-ol	tr		1496	Viridiflorene	0.4
1144	Camphor	17.1		1502	α-Muurolene	0.5
1147	Camphene hydrate	0.5		1514	β-Curcumene	tr
1156	Isoborneol	3.6		1515	γ-Cadinene	1.1
1162	Pinocarvone	0.1		1534	*trans*-Cadina-1,4-diene	tr
1165	Borneol	0.4		1537	α-Cadinene	0.1
1176	Terpinen-4-ol	0.2		1545	α-Calacorene	0.4
1183	*p*-Methylacetophenone	tr		1551	Elemol	tr
1185	Cryptone	0.5		1553	(*Z*)-Caryophyllene oxide	0.2
1190	α-Terpineol	0.7		1567	(*E*)-Nerolidol	0.2
1195	Myrtenal	0.1		1569	Palustrol	0.8
1207	*trans*-Piperitol	0.1		1580	Spathulenol	1.3
1218	*trans*-Carveol	0.1		1584	Caryophyllene oxide	3.7
1225	Bornylformate	0.1		1595	Viridiflorol	14.5
1232	Isobornylformate	0.6		1605	Ledol	0.9
1237	Cuminaldehyde	0.1		1610	Humulene epoxide II	1.5
1241	Carvone	0.1		1616	1,10-di-*epi*-Cubenol	0.4
1245	Thymoquinone	tr		1629	1-*epi*-Cubenol	0.9
1256	Piperitone	23.7		1638	Caryophylla-4(12),8(13)-dien-5β-ol	0.3
1286	Bornyl acetate	0.1		1642	τ-Cadinol	1.3
1291	*p*-Cymen-7-ol	tr		1644	τ-Muurolol	0.9
1293	Thymol	0.1		1647	α-Muurolol (=Torreyol)	0.5
1302	4-Hydroxy-*p*-menth-1-en-3-one	0.5		1656	α-Cadinol	1.9
1316	4-Hydroxycryptone	0.1		2285	Unidentified diterpenoid	2.0
	**TOTAL IDENTIFIED = 97.4%**					

RI: “Retention Index”, determined with respect to a homologous series of *n*-alkanes on an HP-5ms column.

**Table 2:** Antimicrobial activity, *IC*_50_ (μg/mL) of the essential oil from *Piper aduncum* L. collected in Havana, Cuba.

**Bacteria**		
	***E. coli***	***S. aureus***
*Piper aduncum* oil	>64	18.2
Reference drug	0.8	8.3
**Fungi**		
	***C. albicans***	
*Piper aduncum* oil	>64	
Reference drug	2.0	

*IC*_50_: Median inhibitory concentration. Reference drugs: Chloramphenicol for *E. coli,* erythromycin for *S. aureus* and miconazole for *C. albicans.*

**Table 3:** Antiprotozoal activity, *IC*_50_ (μg/mL) of the essential oil from *Piper aduncum* L. collected in Havana, Cuba.

**Sanguine Protozoa**			
	***P. falciparum***	***T. brucei***	***T. cruzi***
*Piper aduncum* oil	1.3	2.0	2.1
Reference drug	0.02	0.04	3.2
***Leishmania* spp.**			
	***L. amazonensis***	***L. donovani***	***L. infantum***
*Piper aduncum* oil	23.8	7.7	8.1
Reference drug	0.02	0.03	3.7

*IC*_50_: Median inhibitory concentration. Reference drugs: Chloroquine for *P. falciparum*, suramine for *T. brucei,* benznidazol for *T. cruzi,* miltefosine for *L. infantum*, and amphotericin B for *L. amazonensis* and *L. donovani*.

**Table 4:** Pharmacological reports of essential oil from *Piper aduncum* L. according to geographical location.

Country	Pharmacological Activities (Description)	Reference
**Brazil**	Acaricidal activity against *Rhipicephalus microplus*	[50]
	Acaricidal activity against *Tetranychus urticae*	[23]
	Insecticidal effect against *Solenopsis saevissima*	[8]
	Insecticidal effect against *A. aegypti*	[51]
	Insecticidal effect against *Diaphorina citri*	[52]
	Insecticidal effect against *Euschistus heros*	[53]
	Antiprotozoal activity against *L. amazonensis* Antibacterial activity against *Mycobacterium tuberculosis*	[43]
	Antiprotozoal activity against *T. cruzi*	[5]
**Cuba**	Antioxidant activity	[7]
	Anthelminthic activity against *Haemonchus contortus*	[6]
	Antimicrobial activity against *S. aureus, P. falciparum, T. cruzi, T. brucei, L. amazonensis* and *L. infantum*	[54]
**Malaysia**	Insect repellent against *A. albopictus*	[55]
	Insecticidal effect against *Periplaneta americana*	[56]
	Insect repellent against *A. aegypti and A. albopictus*	[57]
	Insect repellent against *A. aegypti*	[9]

**Table 5:** Major chemical constituents reported for essential oils of *Piper aduncum* L.

Major components	Pharmacological Activities ^a^	Country	Reference
1,8-Cineole	-	Brazil	[28]
Apiole	-	New Guinea	[21]
Bicyclogermacrene	-	Brazil	[43]
Piperitone	-	Cuba	[7,54]
Dill apiole	Antifungal	Brazil	[25]
	Insecticidal	Brazil	[8,25,52]
	Anti-inflammatory	Brazil	[58]
	Genotoxic	Brazil	[59]
Linalool	Trypanocidal	Brazil	[5]
Nerolidol	Trypanocidal	Brazil	[5]
Safrole	Anti-inflammatory	Brazil	[58]

^a^ Pharmacological activity was only included if pure compound was tested.
